# DuaST: an integrated deep learning framework for spatial transcriptomics with cross-branch interaction

**DOI:** 10.1093/bib/bbag174

**Published:** 2026-04-15

**Authors:** Xiao Liang, Pei Liu, Juping Li, Cong Shen, Jie Cai, Jiawei Luo

**Affiliations:** College of Computer Science and Electronic Engineering, Hunan University, Lushan Southern Road, Changsha 410082, Hunan, China; College of Computer Science and Electronic Engineering, Hunan University, Lushan Southern Road, Changsha 410082, Hunan, China; College of Computer Science and Electronic Engineering, Hunan University, Lushan Southern Road, Changsha 410082, Hunan, China; Academy of Mathematics and Systems Science, Chinese Academy of Sciences, No. 55, Zhongguancun East Road, Beijing 100190, Beijing, China; College of Computer Science and Electronic Engineering, Hunan University, Lushan Southern Road, Changsha 410082, Hunan, China; College of Computer Science and Electronic Engineering, Hunan University, Lushan Southern Road, Changsha 410082, Hunan, China; Yuelushan Laboratory, Hongqi Road, Changsha 410128, Hunan, China

**Keywords:** spatial transcriptomics, deep learning, dual-branch framework, spatial multi-omics, spatially variable genes

## Abstract

Spatial transcriptomics (ST) enables the joint characterization of gene expression and spatial information, indicating the need for generalizable computational methods to exploit these data. However, integrating spatial and non-spatial information remains a challenge. In this study, we propose DuaST, an integrated dual-branch learning framework for ST. Specifically, DuaST designs a spatially aware branch and a non-spatial branch to separately model neighborhood dependencies and topology-agnostic features. To integrate these complementary representations, DuaST employs a synergistic combination of local–global contrastive learning, adversarial alignment, and attention-based fusion. These mechanisms reinforce cross-branch interactions and enable the learning of biologically meaningful representations. Beyond pattern modeling, DuaST identifies spatially variable genes (SVGs) by reconstructing gene expression with a learnable weight matrix that integrates both spatial and non-spatial dependencies. Furthermore, DuaST extends seamlessly from single- to multi-omics analyses, offering a unified framework that leverages supplementary omics to enhance biological insight. The experimental results indicate that DuaST achieves superior performance in several tasks, such as spatial domain identification, SVGs detection, and multi-omics integration. Ablation studies further demonstrate the overall effectiveness of the model design in capturing spatial and non-spatial representations.

## Introduction

The rapid advancement of spatial transcriptomics (ST) technologies has created new avenues for investigating gene expression within the native tissue context. By preserving spatial information, ST facilitates the study of diverse spatial patterns in gene expression. Building on these advances, recent technologies such as 10$\times $ Visium [1], STARmap [2], osmFISH [3], and Stereo-seq [4] have been widely applied to generate spatially resolved gene expression data, thereby elucidating tissue architecture and advancing research across diverse fields [5, 6]. However, many modeling approaches developed for single-cell RNA-seq often omit spatial coordinates [7, 8], limiting comprehensive tissue-level analysis. Collectively, these considerations indicate the need for general computational frameworks capable of analyzing multi-platform ST data.

Several statistical methods [9, 10] attempt to capture the properties of ST data, yet they typically rely on distributional assumptions. Recently, deep learning methods have been applied to model ST data, differing in how they integrate spatial and gene expression information. Variational autoencoder-based approaches, including SEDR [11], AttentionVGAE [12], and spaVAE [13], learn representations while explicitly modeling spatial dependencies, for instance by combining Gaussian process priors with standard priors. Multi-view integration methods, such as MAFN [14], STMGCN [15], and spaGRA [16], adopt distinct strategies, including dynamic feature fusion, graph-based representation learning, or contrastive learning to capture multi-level spot associations. However, they often inadequately leverage spatial and non-spatial information, limiting the effective use of the ST data.

Meanwhile, developing computational methods for accurate spatially variable genes (SVGs) identification has become essential for fully leveraging ST data, as their non-random expression patterns reveal cellular states, intercellular communication, and tissue functions, forming the basis for spatial domain analysis and functional enrichment [17, 18]. Several methods have been developed to identify SVGs, adopting distinct strategies. Gaussian process-based approaches, such as nnSVG [19] and SpatialDE [20], decompose gene expression variability into different components. Statistical scoring methods, including SINFONIA [21] and PROST [22], leverage spatial autocorrelation metrics or integrate statistical significance with expression separability to quantitatively evaluate spatial patterns. Graph-based approaches, exemplified by SpaGCN [23] and STAMarker [24], capture the interdependence between spatial domains and SVGs, facilitating domain-guided identification. Despite these advances, existing methods often do not fully leverage both spatial and non-spatial information, limiting their ability to accurately identify SVGs and their complex, multi-scale expression patterns.

In parallel, the emergence of multi-omics platforms, such as SPOTS [25], spatial-CITE-seq [26], spatial-epigenome-transcriptome [27], and Stereo-CITE-seq [28], enables the simultaneous acquisition of transcriptomics, antibody-derived tag (ADT), and chromatin accessibility (ATAC) data from the same tissue slice. Multi-omics integration has gained increasing attention as a strategy to capture tissue complexity [29, 30], a trend exemplified by recent computational frameworks. For example, PRAGA [31] employs dynamic graphs and prototype-based contrastive learning to enhance feature representation. SpatialGlue [32] leverages graph neural networks with attention to unify spatial and omics features into spot-level representations. SMMGCL [33] constructs hybrid adjacency graphs across modalities and enforces representation consistency via contrastive learning. Despite their advances, these frameworks often rely on complex architectures, which can substantially increase computational burden.

In this work, we present DuaST, a versatile and extensible deep framework for comprehensive ST analysis. To jointly model spatial and non-spatial patterns, DuaST employs a dual-branch representation learning (DBRL) architecture: one branch leverages variational graph auto-encoders (VGAEs) to capture local spatial dependencies and global feature distributions, while the other branch adopts a multilayer perceptron (MLP) to learn non-spatial expression patterns. To coherently integrate these complementary representations, DuaST incorporates three cross-branch interactive strategies: a local–global contrastive mechanism (LGCM) to reinforce multi-scale feature consistency, an adversarial alignment mechanism (AAM) to facilitate modality-invariant representation learning, and an attention fusion mechanism (AFM) to dynamically integrate information across branches. Beyond representation learning, DuaST introduces a spatial relevance scoring (SRS) module that detects SVGs by reconstructing gene expression through a learnable gene-wise weight matrix. Moreover, this framework is modality-flexible, accommodating additional omics without requiring architectural modifications. In multi-omics contexts, the non-spatial branch (NSB) directly accepts other omics as input without modeling their spatial dependencies, resulting in a minimal model design that preserves both spatial and non-spatial information while capturing distinct signals. Extensive evaluations across diverse ST platforms demonstrate that DuaST consistently outperforms state-of-the-art baselines in tasks such as spatial domain identification and SVGs detection.

## Materials and methods

### Overview of DuaST

**Figure 1:**
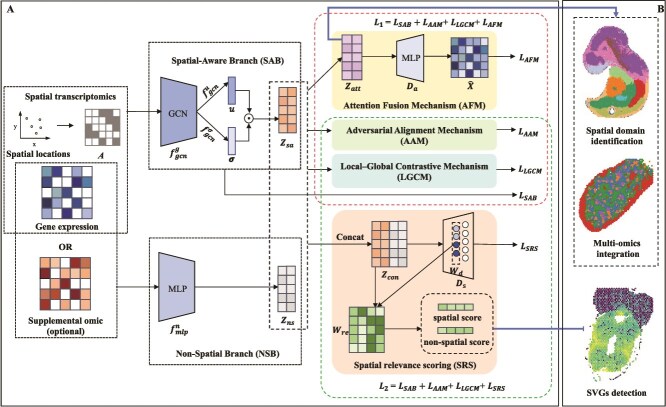
The overall framework of DuaST. A. Dual-branch framework. The SAB captures neighborhood dependencies through graph-based encoding, and the NSB extracts topology-agnostic features, providing two complementary views of the data that are subsequently coordinated through contrastive, adversarial, and attention-based interactions. While SRS operates by reconstructing gene expression with a learnable gene-wise weight matrix, leveraging contrastive and adversarial learning yet without involving the attention mechanism. B. Downstream tasks. DuaST’s tasks in spatial domain identification, multi-omics integration, and SVGs detection.

DuaST is a unified framework for ST analysis that integrates spatial structure with complementary non-spatial information. It takes gene expression and spatial coordinates as inputs, with optional multi-omics modalities. On this basis, DuaST employs a DBRL strategy: the spatial-aware branch (SAB) and the NSB. To effectively integrate these complementary representations, DuaST introduces three cross-branch interaction strategies: LGCM, AAM, and AFM. The resulting unified representations support downstream tasks, such as spatial domain identification and SVG detection. The workflow of DuaST is illustrated in Fig. 1.

### Datasets and preprocessing

DuaST employs a dual-branch architecture to process two feature matrices, ${X_{1}}\in{R}^{{N}\times{d_{1}}}$ and ${X_{2}}\in{R}^{{N}\times{d_{2}}}$, where $N$ is the number of spots or cells, and $d_{1}$, $d_{2}$ denote the respective feature dimensions. This architecture supports both single-omics and multi-omics applications: in single-omics, $X_{1}$ and $X_{2}$ are identical gene expression matrices, whereas in multi-omics (e.g. spatial epigenome–transcriptome), they correspond to gene and ATAC features, respectively. Preprocessing of $X_{1}$ involves standard filtering and normalization, including removal of lowly expressed genes, selection of highly variable genes, and log-transformation with SCANPY [34]. For alternative modalities represented by $X_{2}$, preprocessing follows the SpatialGlue pipeline [32].

We conduct experiments on seven public datasets to verify the effectiveness of the proposed method: (i) human breast cancer (HBC) 10$\times $ Visium data. (ii) E9.5 Mouse Embryo (E9.5) Stereo-seq dataset. (iii) human lymph node (HLN) 10$\times $ Visium dataset. (iv) mouse brain (MB) spatial-epigenome-transcriptome dataset. (v) mouse spleen (MS) SPOTS dataset. (vi) human tonsil (HT) spatial-CITE-seq dataset. (vii) zebrafish melanoma (ZM) 10$\times $ dataset. Additional details on the evaluation metrics and comparative methods are provided in Supplementary Notes S1 and S2.

### Dual-branch representation learning

To simultaneously capture spatially structured and spatially independent signals, DuaST adopts a DBRL strategy, comprising a SAB and a NSB. The core principle of DuaST is to disentangle complementary spatial and non-spatial signals into separate branches, align them softly in a shared latent space without collapsing modality-specific structure, and adaptively recombine them via attention for downstream tasks. The SAB leverages graph-based modeling to characterize local neighborhood dependencies, whereas the NSB captures complementary topology-agnostic or modality-specific features. Their fusion is adaptively weighted by an attention mechanism that preserves signals where informative. This design allows the model to disentangle complementary aspects of the data while preserving architectural flexibility for both single-omics and multi-omics contexts.

#### Spatial-aware branch

A spatial graph $G=(X_{1}, A)$ is first constructed to model spatial relationships among spots. Euclidean distances are calculated from the spot coordinates, and for each spot $i$, its $\mathit{K}$-nearest neighbors (KNNs) are identified. A binary adjacency matrix $A \in R^{N \times N}$ is subsequently defined such that $A_{ij}=A_{ji}=1$ if spot $j$ belongs to the KNNs of spot $i$, and $A_{ij}=0$ otherwise. The spatial graph structure is defined by the resulting adjacency matrix $A$ in conjunction with the gene expression matrix $X_{1}$. The SAB then adopts the VGAE to captures spatial context within this graph, which preserves spatial topology and facilitates the learning of robust representations. VGAE enables probabilistic representation learning by modeling uncertainty and regularizing latent distributions, whereas the Graph Convolutional Network (GCN) captures spatial dependencies through neighborhood aggregation.

Formally, SAB first employs a single-layer GCN encoder $f_{gcn}^{g}$ to generate spatially smoothed gene representations through the aggregating of neighborhood information:


(1) \begin{align*}& {H_{1}}=f_{gcn}^{g}(G) \in{R}^{{N}\times{d_{3}}},\end{align*}

then two parallel GCNs $f_{gcn}^{\mu }$, $f_{gcn}^{\sigma }$ are applied to estimate the mean vector $\mu $ and the log-variance vector $\log \sigma ^{2}$ of the Gaussian parameters, respectively:


(2) \begin{align*}&\begin{array} {c}{\mu = f_{gcn}^{\mu}(H_{1}, A)}, {\quad \sigma = f_{gcn}^{\sigma}(H_{1}, A)}. \end{array}\end{align*}

The latent representation $Z_{sa}\in{R}^{{N}\times{d_{4}}}$ is then sampled using the reparameterization trick:


(3) \begin{align*}&\begin{array} {c}{Z_{sa} = \mu + \sigma \odot \epsilon}, {\quad \epsilon \sim \mathcal{N}(0, I)}, \\ \end{array}\end{align*}

where $\epsilon $ denotes random noise sampled from a standard normal distribution and $\odot $ indicates the element-wise product.

The model is trained by optimizing the evidence lower bound (ELBO):


(4) \begin{align*}& \mathcal{L}_{\mathrm{SAB}} = \mathcal{L}_{\mathrm{BCE}}\big(A, \hat{A}\big) - \mathcal{L}_{\mathrm{KL}}\big(q(Z_{sa}| \mu, \sigma^{2}) \| p(Z_{sa})\big),\end{align*}

where $\hat{A}$ is the reconstructed adjacency matrix obtained through the inner product of representations, i.e. $\hat{A} = \sigma (Z_{sa} Z_{sa}^\top ) \in{R}^{{N}\times N}$. The first term corresponds to the graph reconstruction loss, computed via binary cross-entropy to preserve spot connectivity, whereas the second term is the Kullback–Leibler divergence to regularize latent variables toward the standard normal prior. Thus, optimizing the ELBO enables the model to learn biologically meaningful graph representations while mitigating overfitting.

#### Non-spatial branch

The NSB captures non-spatial patterns beyond graph topology via a two-layer MLP encoder $f_{mlp}^{n}$. The resulting latent representation $Z_{ns}\in{R}^{{N}\times{d_{4}}}$ is defined as follows:


(5) \begin{align*}& \begin{array} {c}Z_{ns} = H_{2}^{L} = f_{mlp}^{n}(X_{2}) = \phi_{L} \circ \cdots \circ \phi_{i} \circ \cdots \circ \phi_{1} (X_{2}), \\ \\ H_{2}^{i} = \phi_{i}(H_{2}^{i-1}) = \mathrm{ReLU}^{i}\big({BN}^{i}(W^{i} H_{2}^{i-1} + b^{i})\big), \end{array}\end{align*}

where $H_{2}^{i}$ denotes the embedding of the $i$th layer, while $W^{i}$ and $b^{i}$ are the learnable weight matrix and bias vector of the $i$th layer, respectively. BN refers batch normalization, and Rectified Linear Unit (ReLU) is the rectified linear activation. This design enhances the complementarity between the spatial and non-spatial representations while maintaining efficiency through the lightweight MLP.

### Cross-branch interaction strategies

Although the dual-branch architecture facilitates complementary encoding, effective integration requires explicit cross-branch constraints. Consequently, DuaST incorporates three synergistic mechanisms to strengthen cross-branch interactions and produce biologically robust latent features.

#### Local–global contrastive mechanism

Inspired by GraphST [35], and building upon Deep Graph Infomax [36] as a concrete implementation, the LGCM maximizes mutual information between spatial and non-spatial representations by contrasting local and global representations to reinforce multi-scale consistency. For each spot $i$, a local summary embedding $c_{i}$ is computed as the average of its neighbors’ representations:


(6) \begin{align*}& {C}_{i} = \frac{1}{|\mathcal{N}(i)|} \sum_{j \in \mathcal{N}(i)} {Z}_{{sa}, j},\end{align*}

where $\mathcal{N}(i)$ denotes the neighbor set of spot $i$. Positive pairs are formed between ${Z}_{{sa}, i}$ and its local summary $C_{i}$, while negative samples ${Z}_{{neg}, i}$ are generated by randomly permuting $Z_{ns}$. This ensures distributional consistency between positive and negative pairs while removing true spatial–non-spatial correspondence. A bilinear discriminator $D_{c}$ is employed to compute the similarity of embedding pairs, and the local contrastive loss is defined as:


(7) \begin{align*} \mathcal{L}_{{local}} = - \frac{1}{N} \sum_{i=1}^{N} \big[ &\log \sigma\big(D_{c}({Z}_{{sa}, i}, {C}_{i})\big) \notag\\ &+ \log \big(1 - \sigma\big(D_{c}({Z}_{{sa}, i}, {Z}_{{neg}, i})\big)\big) \big].\end{align*}

Meanwhile, global-level semantic alignment is enforced by contrasting $Z_{ns}$ with the graph-based representations $Z_{sa}$. The global contrastive loss is formulated as:


(8) \begin{align*} \mathcal{L}_{{global}} = - \frac{1}{N} \sum_{i=1}^{N} \big[ & \log \sigma\big(D_{c}({Z}_{{sa}, i}, {Z}_{{ns}, i})\big) \notag\\ &+ \log \big(1 - \sigma\big(D_{c}({Z}_{{sa}, i}, {Z}_{{neg}, i})\big)\big) \big].\end{align*}

The shared discriminator is used for both local and global contrastive objectives to maintain a consistent similarity metric across scales and reduce model complexity. Finally, both local and global losses are combined to promote multi-scale representation consistency:


(9) \begin{align*}& \mathcal{L}_{\mathrm{LGCM}} = \mathcal{L}_{{local}} + \mathcal{L}_{{global}}.\end{align*}

#### Adversarial alignment mechanism

To facilitate the learning of representations that can be mapped into a shared latent space, DuaST incorporates an adversarial training strategy between $Z_{sa}$ and $Z_{ns}$. This approach is based on the principle of adversarial domain adaptation, in which a discriminator seeks to differentiate between the two branches while the generator is guided to generate embeddings progressively aligned within a shared representation space. The dual-branch encoder, encompassing a SAB and a NSB, is regarded as the generator within the adversarial framework. In particular, a gradient reversal layer (GRL) is employed, which multiplies the gradient by a negative scaling factor during backpropagation. The adversarial principle underlying our approach is inspired by prior studies, CycleGAN [37] for unpaired image-to-image translation and sciCAN [38] for single-cell integration. Building on this foundation, DuaST implements the mechanism in ST, where the discriminator $D_{m}$, realized as an MLP, estimates the probability that an embedding originates from either the spatial-aware or NSB. The adversarial objective is defined as:


(10) \begin{align*}& \mathcal{L}_{\mathrm{AAM}} = - E_{Z_{{sa}}{{}}} \log D_{m}(Z_{{sa}}) - E_{Z_{{ns}}{{}}} \log \left( 1 - D_{m}(Z_{{ns}}) \right),\end{align*}

where $E[\cdot ]$ denotes the expectation over the empirical distribution, and the GRL applies a negative scaling factor during backpropagation to enable adversarial training.

#### Attention fusion mechanism

The AFM integrates the spatial-aware and non-spatial representations an through attention mechanism to adaptively combine $Z_{sa}$ and $Z_{ns}$. This facilitates the dynamic weighting of complementary signals and the generation of a unified embedding optimized for downstream analyses. Specifically, the representations $Z_{sa}$ and $Z_{ns}$ are initially stacked into $Z_{stack} \in R^{N \times 2 \times d_{4}}$, and a lightweight attention network $f_{att}$ is employed to compute branch-wise weights as follows:


(11) \begin{align*}& (\alpha_{1},\alpha_{2}) = \mathrm{softmax}\big(f_{att}(Z_{stack})\big) \in R^{N \times 2},\end{align*}

where $f_{att}$ is implemented as a one-layer linear function projecting the concatenated representations into a scalar per branch. Subsequently, the fused embedding $Z_{att} \in R^{N \times d_{5}}$ is then obtained as:


(12) \begin{align*}& Z_{att} = \alpha_{1} \cdot Z_{sa} + \alpha_{2} \cdot Z_{ns}, \alpha_{1}(i) + \alpha_{2}(i) = 1,\end{align*}

where $\alpha _{1}(i)$ and $\alpha _{2}(i)$ are the weight values of the spatial-aware and non-spatial representations for spot $i$. Based on $Z_{att}$, we apply the mclust [39] to infer spatial clusters from the spot representations (Supplementary Note S3 for details). This unified representation $Z_{att}$ is further decoded by a two-layer MLP $D_{a}$ to reconstruct the gene expression matrix $\hat{X} = f_{mlp}^{A}(Z_{att}) \in R^{N \times d_{1}}$. The reconstruction loss for gene expression is defined using the mean squared error as:


(13) \begin{align*}& \mathcal{L}_{\mathrm{AFM}} = \lVert \hat{X} - X \rVert_{2}^{2}.\end{align*}

Finally, the overall training objective is formulated as a weighted combination of all loss components:


(14) \begin{align*}& \mathcal{L}_{{1}} = \lambda_{1}\,\mathcal{L}_{\mathrm{SAB}} + \lambda_{2}\mathcal{L}_{\mathrm{LGCM}} + \lambda_{3}\,\mathcal{L}_{\mathrm{AAM}} + \lambda_{4}\,\mathcal{L}_{\mathrm{AFM}},\end{align*}

where $\lambda _{1}$, $\lambda _{2}$, $\lambda _{3}$, and $\lambda _{4}$ are trade-off coefficients that balance the contributions of individual objectives. The optimal values (Supplementary Table S1) for these parameters are determined via a grid search procedure. Through this joint optimization, DuaST preserves spatial topology, enforces cross-modality alignment, and strengthens multi-scale feature consistency. These capabilities yield robust and biologically meaningful representations that support downstream tasks such as spatial domain identification, gene expression enhancement, and multi-omics integration. All components are optimized jointly from the first epoch, with gradients flowing through both the spatial and NSBs simultaneously. This end-to-end coupling ensures that the two branches interact throughout training via shared loss objectives, rather than being learned in isolation.

### Spatial relevance scoring

To systematically identify SVGs, we develop the SRS method inspired by spaVAE [13]. By concatenating the spatial $Z_{sa}$ and the non-spatial $Z_{ns}$ representations, we obtain the combined representation $Z_{con} = \mathrm{concat}(Z_{sa}, Z_{ns}) \in R^{N \times 2d_{4}}$. The reconstructed expression matrix is then obtained via a linear decoder $D_{s}$, i.e. $\hat{X}_{c}=D_{s}(Z_{con})=Z_{con}\cdot W_{d}\in R^{N \times d1}$, where $W_{d}$ is a learnable weight matrix. The reconstruction loss $\mathcal{L}_{\mathrm{SRS}} = \lVert \hat{X}{c} - X \rVert _{2}^{2}.$

In this stage, the overall loss function is slightly modified. Unlike the previous design, the attention fusion mechanism $\mathcal{L}_{\mathrm{AFM}}$ is not involved at this stage, and $\mathcal{L}_{\mathrm{SRS}}$ is incorporated instead:


(15) \begin{align*}& \mathcal{L}_{{2}} = \beta_{1}\,\mathcal{L}_{\mathrm{SAB}} + \beta_{2}\mathcal{L}_{\mathrm{LGCM}} + \beta_{3}\,\mathcal{L}_{\mathrm{AAM}} + \beta_{4}\,\mathcal{L}_{\mathrm{SRS}},\end{align*}

where $\beta _{1}$, $\beta _{2}$, $\beta _{3}$, and $\beta _{4}$ denote trade-off coefficients that regulate the relative contributions of the individual objectives. The optimal values of these parameters are obtained by grid search and are also presented in Supplementary Table S1. The SRS for identifying SVGs is a post-hoc analysis conducted after the main model training converges. All model components are frozen, and gene-wise spatial relevance is quantified by decomposing the contribution of the spatial and non-spatial latent subspaces through the trained decoder.

The steps for identifying SVGs are as follows:

- To evaluate the contribution of each dimension to gene-expression reconstruction, we reweight $W_{d}$ by the operation $W_{re} = \mathrm{diag}(Z_{re}) \cdot W_{d}$ to enhance the influence of the most informative dimensions, where $Z_{re}$ is the L2-norm of $Z_{con}$.
- To better capture the relative importance of spatial and non-spatial features, the reweighted matrix $W_{re}$ is first partitioned into two subspaces: a spatially dependent subspace (first half of dimensions) and a spatially agnostic subspace (second half). Then they are averaged separately, i.e. $w_{sa} = \mathrm{avg}(W_{re}[:d_{1}/2,:])$, $w_{ns} = \mathrm{avg}(W_{re}[d_{1}/2:,:])$.
- For each gene, a spatial relevance score is defined as the ratio between the mean weights of the above two subspaces, followed by min–max normalization to scale the scores into the range $[0,1]$. This final score reflects the relative spatial contribution of each gene and enables the systematic identification of SVGs.

## Results

### DuaST improves spatial domain identification and gene expression enhancement

**Figure 2:**
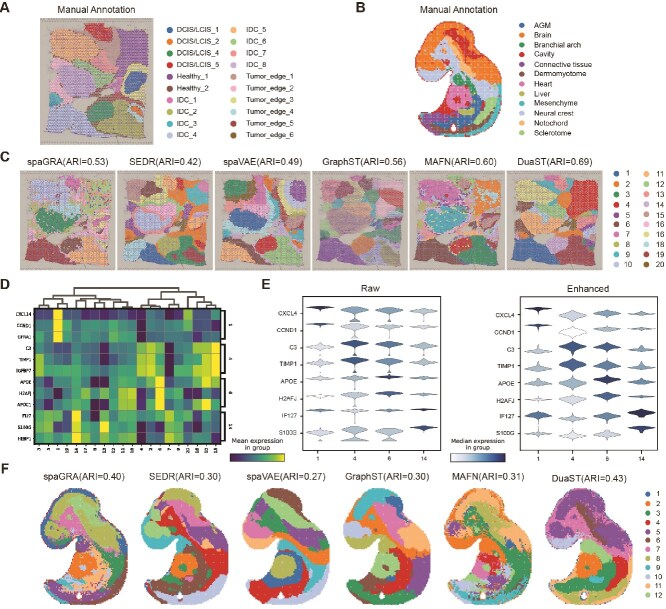
Applied DuaST to the HBC 10$\times $ Visium data and the E9.5 Stereo-seq dataset. (A) The annotation of the HBC dataset. (B) The annotation of the E9.5 dataset. (C) Domain identification by all methods of HBC dataset. (D) The heatmap of the expression of the domains on the top 3 DEGs from partial domains of HBC dataset. (E) The violin plot of raw and enhanced gene expression of HBC dataset. (F) Domain identification by all methods of the E9.5 dataset.

We first assessed the performance of DuaST across ST datasets derived from diverse technologies, starting with a HBC dataset generated on the 10$\times $ Visium platform. This dataset contains 20 annotated regions (Fig. 2A), including healthy tissue (Healthy), ductal carcinoma in situ (DCIS), invasive ductal carcinoma (IDC), and tumor margin areas (Tumor edge). Compared with competing methods, DuaST exhibited superior spatial domain identification performance, achieving the highest ARI of 0.69 (Fig. 2C). The spatial domains identified by DuaST were more continuous and closely aligned with manual annotations, in contrast to the fragmented clusters produced by other methods. Among the comparative methods, MAFN achieved the second-highest ARI of 0.60, although its domain boundaries were closer to annotations than most alternatives, some regions contained noticeable noise. By comparison, spaGRA and SEDR showed poor performance due to overlapping clusters. These results indicated that the dual-branch architecture contributed to spatial domain identification by jointly modeling spatial and non-spatial features.

To examine the molecular distinctions between cancerous and healthy tissues, we analyzed differential gene expressions (DEGs) across IDC_4 (domain 1), Healthy_1 (domain 4), Tumor_edge_2 (domain 6), and DCIS/LCIS_4 (domain 14), identifying the top 3 genes in each domain (Fig. 2D). Notably, *CCND1*, a key cell cycle regulator whose amplification is associated with aggressive phenotypes and poor prognosis in breast cancer [40], was highly enriched in IDC_4 (domain 1). This same domain also showed specific expression of *CXCL14*, a chemokine reported to suppress tumor growth and metastasis, suggesting potential intra-tumoral heterogeneity or context-dependent roles [41]. These genes were mainly enriched within their respective domains, emphasizing spatial heterogeneity and supporting the effectiveness of DuaST in capturing biologically meaningful patterns across cancerous and healthy tissues. In addition, we use violin plots to compare the raw and imputed expression of these marker genes (Fig. 2E). The plots show that DuaST-imputed expression exhibits stronger enrichment within the expected spatial domains and reduced spurious signal in unrelated regions.

Furthermore, we analyzed the E9.5 Mouse Embryo (E9.5) dataset obtained using Stereo-seq technology, which comprised multiple regions, such as the brain, heart, and liver, to further validate the effectiveness of DuaST (Fig. 2B). The results revealed that spaGRA failed to fully recognize the cavity region. In contrast, DuaST demonstrated improved alignment with the annotations, particularly within the liver region (Fig. 2F). Building on these findings, DuaST demonstrated robust performance, accurately identifying multiple organs and tissues and validating its effectiveness in spatial domain recognition within complex tissue identification.

### DuaST enables integrative multi-omics analysis using quantitative assessment

**Figure 3:**
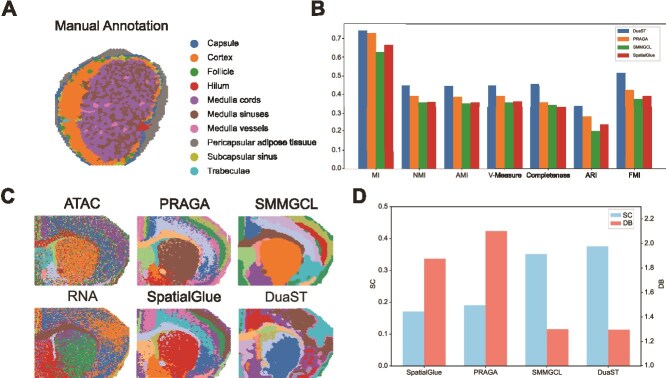
Applied DuaST to the HLN 10$\times $ Visium data and the MB Spatial-epigenome-transcriptome dataset. (A) The annotation of HLN dataset. (B) Performance comparison with baselines using seven metrics of HLN dataset. (C) Visualization of single-omics (ATAC, RNA) and multi-omics integration methods of MB dataset. (D) SC comparison across all methods of MB dataset.

We conducted quantitative evaluations on the HLN dataset generated by 10$\times $ Visium technology that jointly profiles RNA and protein expression, as well as the MB dataset combining RNA-seq and ATAC modalities to integrate transcriptional and epigenomic landscapes (Fig. 3A). Performance was assessed using classical clustering metrics including MI, NMI, AMI, V-Measure, Completeness, ARI and FMI for the HLN dataset with Hematoxylin & Eosin (H&E)-derived ground-truth labels, while the silhouette coefficient (SC) and Davies-Bouldin index (DB) was adopted for the MB dataset lacking reference annotations. Specifically, in the HLN dataset, DuaST surpassed all baseline methods across a wide range of clustering evaluation metrics (Fig. 3B). Moreover, DuaST achieved 2.39% improvement in SC values relative to SMMGCL in the MB dataset and obtained the lowest DB values among all methods (Fig. 3D). The visualizations in the spatial domains and uniform manifold approximation and projection [42] further highlighted the effectiveness of DuaST, showing clearer tissue boundaries and well-separated clusters compared with baseline methods (Supplementary Figs S1 and S2). Collectively, these results demonstrated that DuaST, leveraging its dual-path architecture to capture both spatial expression patterns and cross-omics complementarities, reliably extracts comprehensive representations from spatial multi-omics data.

We further analyzed the MB dataset by separately visualizing RNA-seq and ATAC-seq omics as reference maps (Fig. 3C). Based on these references, DuaST not only recovered the major spatial structures but also distinguished regions where RNA signals were present while ATAC signals were absent, such as the upper-right area. These results highlighted that DuaST, in the context of multi-omics integration, can capture spatial differences that cannot be revealed by single-omics alone.

### DuaST facilitates identification of specific cell types and regions within tissue

We evaluated cell-type composition on the MS dataset produced by the SPOTS platform. The dataset contains RNA and protein features. Based on a preliminary partition generated by SpatialGlue, the tissue was categorized into five major cell types: T cells, B cells, RpMØ (red pulp macrophages), MZMØ (marginal zone macrophages), and MMMØ (marginal metallophilic macrophages). Cell-type identities were assigned based on a combination of spatial patterns and established protein markers.

**Figure 4:**
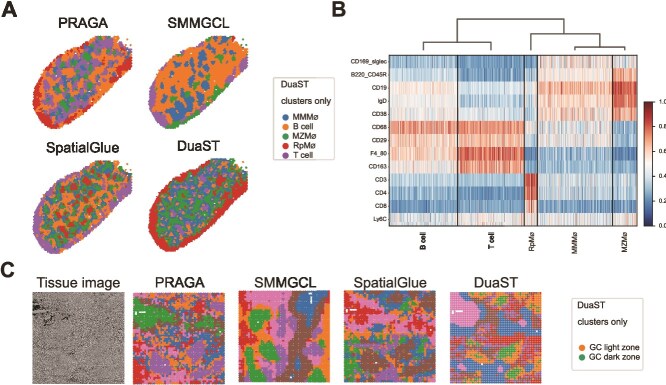
Applied DuaST to the MS SPOTS dataset and the HT spatial-CITE-seq data. (A) Visualization of multi-omics integration methods of MS dataset. (B) Heatmap of the ADTs of MS dataset. (C) Tissue image and visualization of multi-omics integration methods of HT dataset.

While all methods accurately identified the RpMØ, PRAGA and SMMGCL failed to delineate complete boundaries, resulting in mixed regions, and SMMGCL merged MMMØ, B cells, and MZMØ into overlapping clusters, obscuring their spatial separation. The spatial visualization from DuaST showed that RpMØ occupied separate outer regions, B cells were surrounded by T cells, and MMMØ and MZMØ were mixed within and between RpMØ, showing a clear separation of cell types achieved by DuaST (Fig. 4A). A heatmap of cluster-specific proteins was generated to confirm the spatial segregation of cell types observed in the previous analysis, including markers such as CD19 and F4_80 (Fig. 4B).

Next, for the HT tissue dataset, obtained using spatial-CITE-seq to integrate RNA and protein features, we drew on [26] as a methodological reference, partitioned the data into seven clusters, and explicitly distinguished the germinal center (GC) light and dark zones. As shown in Fig. 4C, DuaST produced clustering results with smoother spatial continuity and stronger concordance with the tissue than PRAGA, while SMMGCL failed to distinguish the GC light and dark zones. Collectively, these results demonstrated the ability of DuaST to delineate specific regions within tissue architectures.

### DuaST supports detection of spatially variable genes

**Figure 5:**
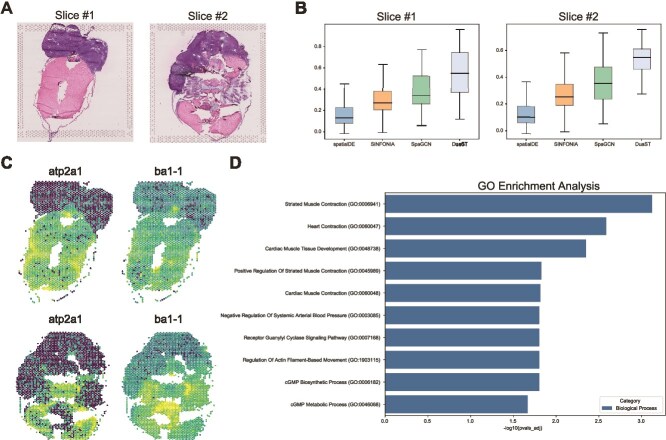
Applied DuaST to the ZM 10$\times $ Visium dataset with two slices. (A) H&E images of ZM on Slices A and B. (B) Boxplots comparing Moran’s I values on Slice A and Slice B. (C) Spatial expression patterns of the selected SVGs atp2a1 and ba1-1. (D) GO enrichment analysis for SVGs.

To assess the capability of DuaST in detecting SVGs, we analyzed ZM slices #1 and #2 (Fig. 5A). Moran’s I values of SVGs detected by each method were compared (Fig. 5B), showing that DuaST achieved higher median Moran’s I values in Slice #1 and Slice #2 (0.55 for both slices). Both SpaGCN and DuaST exhibited significantly higher scores than SINFONIA and spatialDE, demonstrating the benefit of incorporating spatial information. Specifically, DuaST outperformed SpaGCN, highlighting the advantage of its dual-branch architecture in jointly capturing spatial and non-spatial patterns for enhanced SVGs identification.

Figure 5C illustrated the spatial distributions of SVGs selected by DuaST, $\mathit{atp2a1}$ and $\mathit{ba1-1}$, across Slice #1 and Slice #2. These genes showed consistent patterns of enrichment in the same regions across the two slices, demonstrating their reproducibility across tissue sections. In addition, each gene was predominantly enriched in specific regions, with a gradual decrease in expression toward surrounding areas, highlighting their spatial specificity. These results further demonstrated that DuaST can effectively identify SVGs with spatial pattern.

To elucidate the biological significance underlying SVGs identified in ZM dataset, we performed Gene Ontology (GO) enrichment analysis. Using slice #2 as an example, this analysis revealed significant associations with biological processes linked to striated and cardiac muscle contraction, cGMP metabolism, and actin filament regulation (Fig. 5D). These findings suggested that spatial heterogeneity in gene expression may reflect dynamic interactions between melanoma progression and cardiovascular/muscular systems, potentially mediated by metabolic reprogramming or mechanical stress responses. These findings tentatively suggested that spatial heterogeneity in gene expression may reflect dynamic interactions between melanoma progression and cardiovascular/muscular systems, potentially mediated by metabolic reprogramming or mechanical stress responses. However, given the neural crest origin of ZM cells—which retain developmental plasticity toward multiple lineages including cardiac and skeletal muscle precursors [43]—such enrichment could also arise from reactivation of latent embryonic programs rather than direct tumor–tissue crosstalk. The prominence of cardiac-related terms (e.g. GO:0060047, GO:0048738) hinted at either tumor-induced perturbations in adjacent tissues or model-specific features of zebrafish embryonic physiology. Furthermore, enrichment of cGMP signaling pathways implied a role for second messengers in modulating tumor microenvironmental adaptations.

### Ablation study

To further investigate the mechanism of DuaST, we conducted a series of ablation studies using the HBC dataset. Specifically, we removed the AAM (w/o-AAM), the LGCM (w/o-LGCM), the ELBO of the spatial-aware branch (w/o-SAB), and the AFM (w/o-AFM) to assess their individual and combined contributions to model performance. In addition, we further examined the effects of the local contrastive mechanism (w/o-LCM) and the global contrastive mechanism (w/o-GCM) individually to better disentangle their complementary roles in the overall framework.

**Figure 6:**
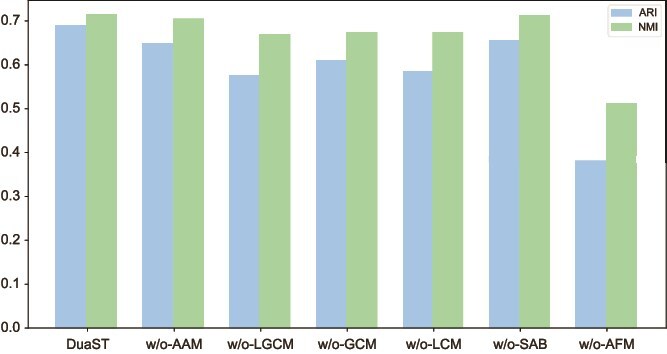
Boxplot of ARI and NMI for DuaST and its variants on HBC dataset.

As shown in Fig. 6, the AFM played a central role in integrating dual-path information, as removing it (w/o-AFM) led to the most pronounced reduction in ARI and NMI. This is because the AFM not only aggregated complementary information from the two branches, but also adaptively emphasizes their most informative features, making the fused representation more discriminative. Other variants also resulted in varying degrees of performance degradation, highlighting the complementary importance of each module. The integration of dual-branch synergistically enhanced DuaST’s ability to effectively fuse spatial information with gene expression, improving its performance in ST analysis.

## Discussion and conclusion

DuaST provides a versatile and extensible framework for the analysis of ST data. The framework collaboratively learns spatial and non-spatial patterns via a dual-branch encoder and utilizes an AFM to dynamically balance these complementary representations. Its modular design permits the incorporation of additional omics modalities, enabling both single-omics and multi-omics analyses without requiring modifications to the underlying architecture. To improve the quality of learned representations, DuaST integrates contrastive and adversarial strategies that enhance embedding consistency and domain alignment, which enables precise spatial domain delineation. In addition, DuaST develops the SRS method for identifying biologically meaningful SVGs, which explicitly integrates both spatial and non-spatial dependencies to capture informative spatial expression patterns. Comprehensive evaluations across diverse ST platforms indicate that DuaST shows improved performance in tasks such as SVGs identification and multi-omics integration. In the future, we will also explore explicit modeling of spatial dependencies in auxiliary modalities, in addition to more comprehensive integration and deeper exploitation of omics modalities, as well as model spatial-temporal dynamics across multiple sections or time points to facilitate inference of developmental or disease-associated processes.

**Key Points**

- We develop DuaST, a unified deep learning-based dual-branch framework that simultaneously captures spatial and non-spatial gene expression patterns.
- DuaST integrates complementary information across branches using contrastive strategy, adversarial strategy, and attention strategy to adaptively combine representations.
- By flexibly incorporating additional omics modalities into the non-spatial branch, DuaST enables minimal yet effective multi-omics integration while simultaneously supporting spatially variable gene detection, without increasing model complexity.
- DuaST demonstrates superior performance across multiple spatial transcriptomics datasets in spatial domain identification, SVG detection, and multi-omics integration.

## Supplementary Material

DuaST_Supplementary_bbag174

## Data Availability

All the datasets analyzed in this study are public. The detailed information of the dataset and the download link are shown in Supplementary Tables S2 and S5 respectively. An open-source Python implementation of the DuaST toolkit is accessible at https://github.com/liangxiao-cs/DuaST.

## References

[1] Ji AL, Rubin AJ, Thrane K et al. Multimodal analysis of composition and spatial architecture in human squamous cell carcinoma. *cell* 2020;182:497–514. 32579974 10.1016/j.cell.2020.05.039 PMC7391009

[2] Wang X, Allen WE, Wright MA et al. Three-dimensional intact-tissue sequencing of single-cell transcriptional states. *Science* 2018;361:eaat5691. 29930089 10.1126/science.aat5691 PMC6339868

[3] Codeluppi S, Borm LE, Zeisel A et al. Spatial organization of the somatosensory cortex revealed by osmfish. *Nat Methods* 2018;15:932–5. 30377364 10.1038/s41592-018-0175-z

[4] Chen A, Liao S, Cheng M et al. Spatiotemporal transcriptomic atlas of mouse organogenesis using DNA nanoball-patterned arrays. *Cell* 2022;185:1777–92. 35512705 10.1016/j.cell.2022.04.003

[5] Moses L, Pachter L. Museum of spatial transcriptomics. *Nat Methods* 2022;19:534–46. 35273392 10.1038/s41592-022-01409-2

[6] Rao A, Barkley D, França GS et al. Exploring tissue architecture using spatial transcriptomics. *Nature* 2021;596:211–20. 34381231 10.1038/s41586-021-03634-9 PMC8475179

[7] Liu Y, Wei G, Jia-Shun W et al. Music-GCN: a novel multi-tasking pipeline for analyzing single-cell transcriptomic data using residual graph convolution network. IEEE Transactions on Computational Biology and Bioinformatics 2025;22:781–9. 40811400 10.1109/TCBBIO.2025.3534776

[8] Wang L, Nie R, Miao X et al. InClust+: the deep generative framework with mask modules for multimodal data integration, imputation, and cross-modal generation. *BMC Bioinform* 2024;25:41. 10.1186/s12859-024-05656-2 PMC10809631 38267858

[9] Shang L, Zhou X. Spatially aware dimension reduction for spatial transcriptomics. *Nat Commun* 2022;13:7203. 36418351 10.1038/s41467-022-34879-1 PMC9684472

[10] Zhao E, Stone MR, Ren X et al. Spatial transcriptomics at subspot resolution with bayesspace. *Nat Biotechnol* 2021;39:1375–84. 34083791 10.1038/s41587-021-00935-2 PMC8763026

[11] Hang X, Huazhu F, Long Y et al. Unsupervised spatially embedded deep representation of spatial transcriptomics. *Genome Med* 2024;16:12. 38217035 10.1186/s13073-024-01283-x PMC10790257

[12] Lei L, Han K, Wang Z et al. Attention-guided variational graph autoencoders reveal heterogeneity in spatial transcriptomics. *Brief Bioinform* 2024;25:bbae173. 38627939 10.1093/bib/bbae173 PMC11021349

[13] Tian T, Zhang J, Lin X et al. Dependency-aware deep generative models for multitasking analysis of spatial omics data. *Nat Methods* 2024;21:1501–13. 38783067 10.1038/s41592-024-02257-y PMC12410143

[14] Zhu Y, He X, Tang C et al. Multi-view adaptive fusion network for spatially resolved transcriptomics data clustering. *IEEE Trans Knowl Data Eng* 2024;36:8889–900.

[15] Shi X, Zhu J, Long Y et al. Identifying spatial domains of spatially resolved transcriptomics via multi-view graph convolutional networks. *Brief Bioinform* 2023;24:bbad278. 10.1093/bib/bbad278 37544658

[16] Sun X, Zhang W, Li W et al. SpaGRA: graph augmentation facilitates domain identification for spatially resolved transcriptomics. *J Genet Genomics* 2025;52:93–104. 39362628 10.1016/j.jgg.2024.09.015

[17] Yan G, Hua SH, Li JJ. Categorization of 34 computational methods to detect spatially variable genes from spatially resolved transcriptomics data. *NatCommun* 2025;16:1141. 10.1038/s41467-025-56080-w PMC11779979 39880807

[18] Li K, Yan C, Chenghao Li L et al. Computational elucidation of spatial gene expression variation from spatially resolved transcriptomics data. *Mol Ther Nucleic Acids* 2022;27:404–11. 35036053 10.1016/j.omtn.2021.12.009 PMC8728308

[19] Weber LM, Saha A, Datta A et al. nnSVG for the scalable identification of spatially variable genes using nearest-neighbor Gaussian processes. *Nat Commun* 2023;14:4059. 37429865 10.1038/s41467-023-39748-z PMC10333391

[20] Svensson V, Teichmann SA, Stegle O. SpatialDE: identification of spatially variable genes. *Nat Methods* 2018;15:343–6. 29553579 10.1038/nmeth.4636 PMC6350895

[21] Jiang R, Li Z, Jia Y et al. SINFONIA: scalable identification of spatially variable genes for deciphering spatial domains. *Cells* 2023;12:604. 36831270 10.3390/cells12040604 PMC9954745

[22] Liang Y, Shi G, Cai R et al. PROST: quantitative identification of spatially variable genes and domain detection in spatial transcriptomics. *Nat Commun* 2024;15:600. 38238417 10.1038/s41467-024-44835-w PMC10796707

[23] Jian H, Li X, Coleman K et al. SpaGCN: integrating gene expression, spatial location and histology to identify spatial domains and spatially variable genes by graph convolutional network. *Nat Methods* 2021;18:1342–51. 34711970 10.1038/s41592-021-01255-8

[24] Zhang C, Dong K, Aihara K et al. STAMarker: determining spatial domain-specific variable genes with saliency maps in deep learning. *Nucleic Acids Res* 2023;51:e103–3. 37811885 10.1093/nar/gkad801 PMC10639070

[25] Ben-Chetrit N, Niu X, Swett AD et al. Integration of whole transcriptome spatial profiling with protein markers. *Nat Biotechnol* 2023;41:788–93. 36593397 10.1038/s41587-022-01536-3 PMC10272089

[26] Liu Y, DiStasio M, Graham S et al. High-plex protein and whole transcriptome co-mapping at cellular resolution with spatial cite-seq. *Nat Biotechnol* 2023;41:1405–9. 36823353 10.1038/s41587-023-01676-0 PMC10567548

[27] Zhang D, Deng Y, Kukanja P et al. Spatial epigenome–transcriptome co-profiling of mammalian tissues. *Nature* 2023;616:113–22. 36922587 10.1038/s41586-023-05795-1 PMC10076218

[28] Liao S, Yang H, Liu W et al. Integrated spatial transcriptomic and proteomic analysis of fresh frozen tissue based on stereo-seq. bioRxiv. 2023. 10.1101/2023.04.28.538364

[29] Vandereyken K, Sifrim A, Thienpont B et al. Methods and applications for single-cell and spatial multi-omics. *Nat Rev Genet* 2023;24:494–515. 36864178 10.1038/s41576-023-00580-2 PMC9979144

[30] Kiessling P, Kuppe C. Spatial multi-omics: novel tools to study the complexity of cardiovascular diseases. *Genome Med* 2024;16:14. 38238823 10.1186/s13073-024-01282-y PMC10795303

[31] Huang X, Ma Z, Meng D et al. PRAGA: prototype-aware graph adaptive aggregation for spatial multi-modal omics analysis. In: *Proceedings of the AAAI Conference on Artificial Intelligence*, Vo. 39, 2025, pp. 326–33. Philadelphia, PA, USA: AAAI.

[32] Long Y, Ang KS, Sethi R et al. Deciphering spatial domains from spatial multi-omics with spatialglue. *Nat Methods* 2024;21:1658–67. 38907114 10.1038/s41592-024-02316-4 PMC11399094

[33] Wang B, Liu W, Luo J et al. SMMGCL: a novel multi-level graph contrastive learning framework for integrating spatial multi-omics data. In: 2024 IEEE International Conference on Bioinformatics and Biomedicine (BIBM), pp. 1213–8. Lisbon, Portugal: IEEE, 2024.

[34] Wolf FA, Angerer P, Theis FJ. SCANPY: large-scale single-cell gene expression data analysis. *Genome Biol* 2018;19:1–5. 29409532 10.1186/s13059-017-1382-0 PMC5802054

[35] Long Y, Ang KS, Li M et al. Spatially informed clustering, integration, and deconvolution of spatial transcriptomics with graphst. *NatCommun* 2023;14:1155. 10.1038/s41467-023-36796-3 PMC9977836 36859400

[36] Velickovic P, Fedus W, Hamilton WL et al. Deep graph infomax. In: *International Conference on Learning Representations (Poster)*, Vol. 2, p. 4, 2019. New Orleans, Louisiana, USA: OpenReview.

[37] Zhu J-Y, Park T, Isola P et al. Unpaired image-to-image translation using cycle-consistent adversarial networks. In: Proceedings of the IEEE International Conference on Computer Vision, pp. 2223–32, 2017. Venice, Italy: IEEE.

[38] Yang X, Begoli E, McCord RP. sciCAN: single-cell chromatin accessibility and gene expression data integration via cycle-consistent adversarial network. *NPJ Syst Biol Appl* 2022;8:33. 36089620 10.1038/s41540-022-00245-6 PMC9464763

[39] Fraley C, Raftery AE. Enhanced model-based clustering, density estimation, and discriminant analysis software: Mclust. *Journal of classification* 2003;20:263–86.

[40] Valla M, Klæstad E, Ytterhus B et al. CCND1 amplification in breast cancer-associations with proliferation, histopathological grade, molecular subtype and prognosis. *J Mammary Gland Biol Neoplasia* 2022;27:67–77. 35459982 10.1007/s10911-022-09516-8 PMC9135839

[41] Xiao-Li G, Zhou-Luo O, Lin F-J et al. Expression of CXCL14 and its anticancer role in breast cancer. *Breast Cancer Res Treat* 2012;135:725–35. 22910931 10.1007/s10549-012-2206-2

[42] Becht E, McInnes L, Healy J et al. Dimensionality reduction for visualizing single-cell data using UMAP. *Nat Biotechnol* 2019;37:38–44. 10.1038/nbt.4314 30531897

[43] Ceol CJ, Houvras Y, Jane-Valbuena J et al. The histone methyltransferase SETDB1 is recurrently amplified in melanoma and accelerates its onset. *Nature* 2011;471:513–7. 21430779 10.1038/nature09806 PMC3348545

