# Supplementary material for: DuaST: an integrated deep learning framework for spatial transcriptomics with cross-branch interaction
Source: Brief Bioinform. 2026 Apr 15;27(2):bbag174. doi: 10.1093/bib/bbag174 (PMC13082397; doi:10.1093/bib/bbag174)
Supplement: DuaST_Supplementary_bbag174 [file duast_supplementary_bbag174.pdf]

Supplementary Materials for

DuaST: An Integrated Deep Learning Framework for Spatial Transcriptomics with Cross-Branch Interaction

Supplementary Notes

Note S1 (Evaluated metrics)

Adjusted Rand Index (ARI)

The Adjusted Rand Index (ARl) ranges from -1 to 1, where higher values signify improved clustering performance. Given the ground truth class labels e' and the predicted class labels e, the ARl can be calculated using the following formula:

$$ARI(e,e') = \frac{\sum_{p,g}\binom{N_{pg}}{2} - \left[\sum_p\binom{N_p}{2}\sum_g\binom{N_g}{2}\right]/\binom{N}{2}}{\frac{1}{2}\left[\sum_p\binom{N_p}{2} + \sum_g\binom{N_g}{2}\right] - \left[\sum_p\binom{N_p}{2}\sum_g\binom{N_g}{2}\right]/\binom{N}{2}}$$

In this context, N represents the total number of spots, N_g denotes the number of spots within the true cluster g, N_p indicates the number of spots in the predicted cluster p, and N_{pg} refers ta the number of spots that are common to both clusters p and g.

Moran's I

Moran's I is a statistical measure that quantifies the degree of spatial autocorrelation within a dataset. For a given gene, Moran's I evaluates how gene expression at a specific location correlates with its surrounding spatial context. When neighboring spots display attraction or repulsion, it indicates spatial dependence, underscoring the presence of spatial autocorrelation in gene expression patterns. Moran's I ranges from -1 to 1, where values near 1 signify strong spatial structure, values around 0 indicate random distribution, and values approaching -1 correspond to an alternating, checkerboard-like pattern. To quantify the spatial heterogeneity of a given gene, Moran's I is computed as follows:

$$I = \frac{N}{W} \frac{\sum_i \sum_j \left[w_{ij}(x_i - \bar{x})(x_j - \bar{x})\right]}{\sum_i (x_i - \bar{x})^2}$$

In this context, x_i and x_j represent the gene expression levels at spots i and j, respectively, while $\bar{x}$ denotes the mean expression across all spots. N indicates the total number of spots, and w_{ij} refers to the spatial weight between spots i and j,calculated based on their 2D spatial coordinates. The term W represents the sum of all w_{ij}.

Moran's I is adopted in this study as the primary quantitative metric for evaluating spatially variable genes. It provides a simple and intuitive measure of spatial expression consistency across neighboring locations by explicitly incorporating spatial neighborhood information.

Silhouette Coefficient (SC)

The SC score quantifies clustering quality by comparing the mean intra-cluster distance (a) with the mean nearest-cluster distance (b) for each sample. The SC score is defined as (b − a)/max(a, b), where a lower value denotes poor clustering, and a higher value, approaching 1, signifies well-separated and compact clusters.

Davies-Bouldin Index (DB)

The DB index evaluates clustering quality based on intra-cluster similarity and inter-cluster separation. For each cluster, the average distance between points within the cluster (intra-cluster dispersion) is compared with the distance to other clusters (inter-cluster separation). The DB score is defined as the average of the maximum similarity values between each cluster and all other clusters. A lower DB value indicates better clustering performance, as it reflects compact clusters that are well separated from each other.

Mutual information（MI)

Mutual information measures the mutual dependence between X and Y. Mutual information measures the mutual dependence between two random variables X and Y. It quantifies the amount of information shared between them.

$$MI(X,Y) = \sum_{i=1}^{|X|} \sum_{j=1}^{|Y|} P(i,j)\, log\, \frac{P(i,j)}{P(i)P(j)}$$

Normalized mutual information (NMI)

Normalized mutual information is a standardized version of mutual information that scales the measure to a fixed range, typically [0,1]. It enables comparison across different datasets or clustering results by accounting for variable entropy levels.

$$NMI(X,Y) = \frac{MI(X,Y)}{H(X)H(Y)}$$

Adjusting the mutual information (AMI)

Adjusting the mutual information corrects for chance by adjusting the expected value of MI under random clustering. This reduces the bias due to randomness and provides a more accurate measure of true cluster similarity.

$$AMI(X,Y) = \frac{MI(X,Y) - \mathbb{E}(MI(X,Y))}{max(H(X),H(Y)) - \mathbb{E}(MI(X,Y))}$$

Fowlkes-Mallows Index (FMI)

FMI measures the similarity between two clustering results by combining intra-class and inter-class pair agreements into a single score. It is defined as the geometric mean of precision and recall based on shared pairs, reflecting both completeness and accuracy of clustering.

$$\mathrm{FMI}(X, Y) = \sqrt{\frac{TP^2}{(TP + FP)(TP + FN)}}$$

V-Measure

V-Measure is the harmonic mean of homogeneity and completeness, balancing the extent to which clusters contain only members of a single class and all members of a class are assigned to the same cluster. It provides a symmetric evaluation of clustering quality independent of cluster labeling.

$$V - Measure(X, Y) = 2 \times \frac{H(X|Y) + H(Y|X)}{H(X) + H(Y)}$$

Completeness

Completeness measures whether all data samples from the same true class are assigned to the same cluster. It evaluates the extent to which the clustering captures class homogeneity.

$$Completeness(X, Y) = 1 - \frac{H(Y|X)}{H(Y)}$$

Note S2 (Comparison with baseline methods)

To assess the spatial domain identification capability of DuaST, we benchmarked it against four methods: spaGRA, SEDR, spaVAE, and MAFN. To evaluate its effectiveness in identifying spatially variable genes (SVGs), we compared it with SpatialDE, SINFONIA, and SpaGCN. In addition, to examine its performance in multi-omics integration, we further benchmarked it against PRAGA, SMMGCL, and SpatialGlue. All methods have been cited in the main text.

Table S3. Single- vs multi-omics baseline methods.

Method	Category	Code
spaGRA	Single-omics	https://github.com/sunxue-yy/SpaGRA
SEDR	Single-omics	https://github.com/JinmiaoChenLab/SEDR
spaVAE	Single-omic	https://github.com/ttgump/spaVAE
MAFN	Single-omics	https://github.com/zhubbbzhu/MAFN
PRAGA	Multi-omics	https://github.com/Xubin-s-Lab/PRAGA
SMMGCL	Multi-omics	https://github.com/cs-wangbo/SMMGCL
SpatialGlue	Multi-omics	https://github.com/JinmiaoChenLab/SpatialGlue

For all baseline methods, hyperparameters were selected using a three-tier strategy without any fine-tuning: (i) if the original authors reported results on the same dataset, their exact settings were used; (ii) otherwise, parameters from a dataset of the same spatial platform (e.g., 10x Visium) were adopted; (iii) if neither was available, default values from the official implementation were used.

Note S3 (Details of Clustering)

Following clustering, we employ an optional refinement strategy to enhance the clustering results. Specifically, if the majority of a given spot's neighboring spots belong to a different domain, its label is reassigned to align with the predominant label of the surrounding domain. This refinement procedure is specifically applied to the HBC dataset.

Note S4 (Hyperparameter Search and Reproducibility)

Hyperparameter selection in DuaST was conducted using a systematic grid search strategy. Specifically, the trade-off coefficients $\lambda1$, $\lambda2$, $\lambda3$, and $\lambda4$, which balance the contributions of different loss components, were tuned independently for each dataset. For all datasets, the same predefined search ranges were used:

$$\lambda1 \in \{1, 5, 10, 20, 30, 40, 50, 60\}$$

$$\lambda2, \lambda3, \lambda4 \in \{0.01, 0.05, 0.1, 0.5, 1, 5, 10\}.$$

The optimal configuration was selected based on task-specific evaluation metrics

reported in the main text. To ensure reproducibility, all experiments were performed with a fixed random seed (2025), and model initialization, data shuffling, and training procedures were kept identical across runs. Once the hyperparameters were determined, the training process became fully deterministic, and repeated runs produced identical results under the same configuration.

Note S5 (Computational Efficiency and Scalability)

To evaluate the computational efficiency and scalability of DuaST, we conducted a profiling analysis on the E9.5 Mouse Embryo dataset (5913 spots and 25568 genes). All experiments were performed on a single NVIDIA GeForce RTX 4090 GPU and repeated five times with 200 training epochs. We report total training runtime and peak GPU memory consumption while progressively increasing the number of spots via subsampling.

As summarized in Table S4, the total runtime increases smoothly with the number of spots, while peak GPU memory usage remains below 1728 MB for the full dataset.

Table S4. Resource usage and scalability on the E9.5 Mouse Embryo dataset.

Spots	Total Runtime (s)	Peak Memory (MB)
1000	9.68	732
2000	10.52	902
3000	11.84	1018
4000	13.36	1264
5000	15.20	1434
5913	17.54	1728

Supplementary Tables

The parameter settings of DuaST

For different datasets, we employ a grid search method to optimize the parameters of DuaST. The specific values for these parameters are provided below.

Table S1. Summary of parameter settings.

Dataset	λ_1	λ_2	λ_3	λ_4
Human breast cancer	20	5	1	0.01
Mouse embryo E9.5	5	1	5	0.05
Mouse Spleen	50	0.5	5	0.5
Human Lymph Node	30	1	0.1	0.01
Mouse Brain	20	1	0.05	10
Human Tonsil	1	10	0.5	10
Zebrafish Melanoma #1	40	0.1	10	0.01
Zebrafish Melanoma #2	10	0.1	0.1	5

Summary of all datasets used in this study

Table S2. Summary of datasets. The "Size" column lists the number of spots for RNA first, followed by other omics.

Platform	Section	Omics	Size (spots x (genes/proteins/adts))
10x Visium	Human breast cancer	RNA	3798*36601
Stereo-seq	Mouse embryo E9.5	RNA	5913*25568
SPOTS	Mouse Spleen	RNA-protein	2568 *32285<br>2568*21
10x Visium	Human Lymph Node	RNA-ADT	3484*18085<br>3484*31
spatial-epigenome-transcriptom	Mouse Brain	ATAC–RNA	9215*22914<br>9215*121068
spatial-	Human Tonsil	RNA-	2492*2000

CITE-seq		protein	2492*283
10x Visium	Zebrafish Melanoma #1	RNA	2179*32268
10x Visium	Zebrafish Melanoma #2	RNA	2677*32268

Table S5. Download link for the dataset

Section	Download link
Human breast cancer	https: //www.10xgenomics.com/datasets/human- breast- cancer-block- a -section-1-1-standard-1-1-0
Mouse embryo E9.5	https://db.cngb.org/stomics/mosta/
Mouse Spleen	https://www.ncbi.nlm.nih.gov/geo/query/acc.cgi?acc=GSE198353
Human Lymph Node	https://www.ncbi.nlm.nih.gov/geo/query/acc.cgi?acc=GSE263617
Mouse Brain	https://web.atlasxomics.com/visualization/Fan/
Human Tonsil	https://www.ncbi.nlm.nih.gov/geo/query/acc.cgi?acc=GSM6578062
Zebrafish Melanoma #1	https://www.ncbi.nlm.nih.gov/geo/query/acc.cgi?acc=GSM4838132
Zebrafish Melanoma #2	https://www.ncbi.nlm.nih.gov/geo/query/acc.cgi?acc=GSM4838133

Supplementary Figures

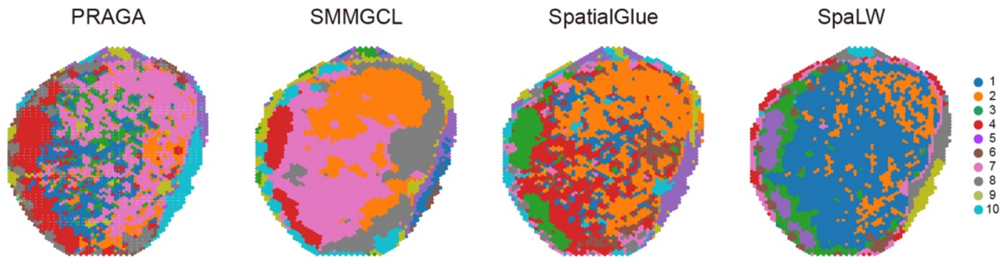

Fig. S1. Domain identification by all methods of HLN dataset.

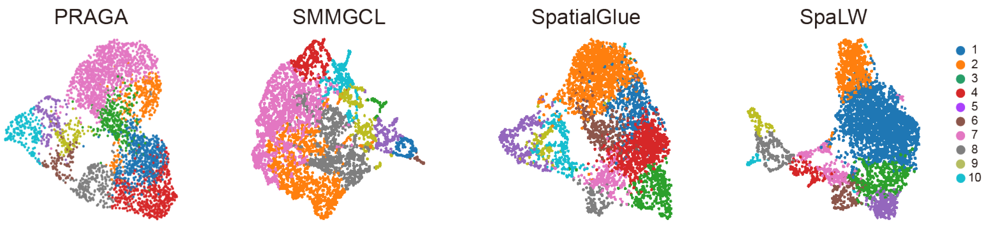

Fig. S2. UMAP visualization generated based on the embedding on the HLN dataset.

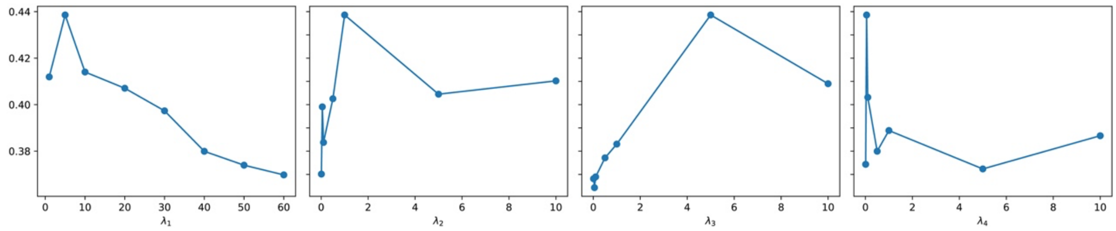

Fig. S3. Local hyperparameter sensitivity analysis of DuaST on the E9.5 Mouse Embryo dataset.
